# In situ structure of the human gap junction

**DOI:** 10.1126/sciadv.aea4183

**Published:** 2026-05-13

**Authors:** Evans Eshriew, Esa-Pekka Kumpula, Shiv K. Sah-Teli, Amiel Abettan, Amina Djurabekova, Vivek Sharma, Juha T. Huiskonen

**Affiliations:** ^1^ Institute of Biotechnology, Helsinki Institute of Life Science HiLIFE, University of Helsinki, 00014 Helsinki, Finland.; ^2^ Department of Physics, University of Helsinki, 00014 Helsinki, Finland.

## Abstract

Gap junction plaques (GJPs) enable direct intercellular communication and consist of connexin channels arranged into two-dimensional lattices. While structures of purified connexin channels have informed models of gating, they omit key intracellular regions and lack native context. Here, we use cryo–electron tomography and focused ion beam milling to determine the in situ structure of human connexin 43 (Cx43) GJPs in HEK293 cells at 14-Å resolution. We reveal a previously unresolved structural contribution of the large carboxyl-terminal domain to lateral channel-channel interactions that appear critical for plaque assembly. Coarse-grained molecular dynamics simulations suggest how lipids and cholesterol occupy the space between adjacent connexins. These findings resolve a decades-old question regarding gap junction organization and highlight a mechanistic function for the carboxyl-terminal domain, likely regulated by a helix-loop-helix motif. Our study provides a structural blueprint for understanding how connexin diversity and regulation shape tissue-level communication in health and disease.

## Teaser

In situ cryo-ET reveals how gap junction channels organize into plaques via the interactions between intracellular domains.

## INTRODUCTION

**Fig. 1.**
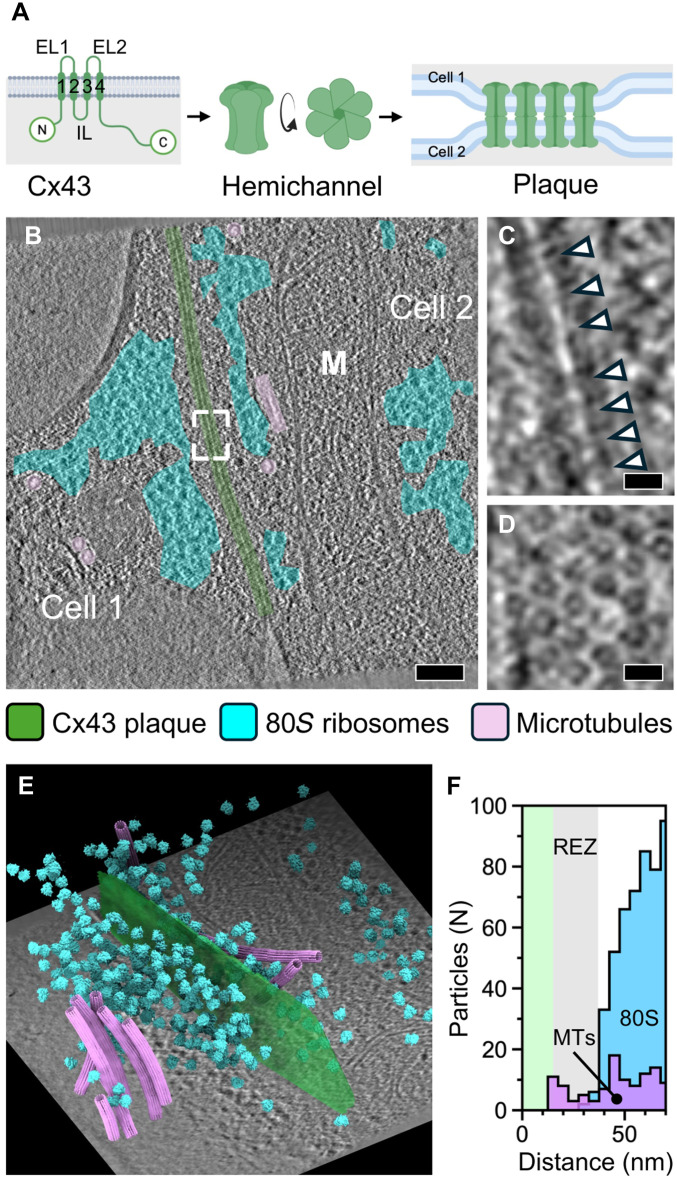
Cryo-ET and template matching of HEK293 Cx43-eGFP cells. (**A**) A schematic representation of the gap junction assembly from connexin 43 (Cx) monomers to hemichannels and to the gap junction plaque between two cells. Intra- (IL) and extracellular (EL) loops are labeled. The transmembrane (TM) helices are numbered 1 to 4. (**B**) One-nm-thick slice through a cryo-ET reconstruction of a cell-cell junction. Areas harboring Cx43 channels (green), 80*S* ribosomes (cyan), and microtubules (pink) have been indicated. A mitochondrion has been labeled (M). Scale bars, 100 nm. (**C**) Close-up of the area indicated in (B). Side views of Cx43 GJCs bridging two plasma membranes are indicated with arrowheads. Scale bar, 10 nm. (**D**) Close-up from a different cryo-ET reconstruction showing top views of the Cx43 GJCs. Scale bar, 10 nm. (**E**) Results of template matching for Cx43 GJCs, 80*S* ribosomes, and microtubule segments are shown after plotting the templates back onto the cryo-ET tomogram (illustrated with a 1-nm-thick slice in the background). The gap junction is depicted as a transparent surface (green) fitted to the Cx43 channel positions for visual clarity. (**F**) Histogram of pairwise distances between 80*S* ribosomes and microtubule segments (MTs) to the Cx43 GJCs, calculated from multiple cryo-ET reconstructions (*n* = 26). The 15-nm-wide zone occupied by Cx43 channels is indicated in green. The 22-nm-wide ribosome exclusion zone (REZ) is indicated in gray.

Gap junctions play a crucial role in intercellular communication by enabling metabolic and electrical coupling at cell-cell contact sites (*1*). Gap junction plaques (GJPs) comprise hundreds of gap junction channels (GJCs) arranged into two-dimensional (2D) lattices that bridge the plasma membranes of adjacent cells (*2*). These channels permit the bidirectional exchange of ions, secondary messengers, hormones, and other small molecules and are involved in diverse processes such as cell differentiation, tumor progression and suppression, and cardiac conduction (*3*). Each GJC is a dodecamer consisting of two hexameric connexin oligomers that dock head-to-head via their extracellular loops (Fig. 1A) (*4*). Of the 21 connexin isoforms identified in humans, each exhibits distinct conductance, permeability, oligomerization compatibility, regulatory mechanisms, and tissue distribution (*5*–*7*). Mutations in connexin genes are linked to diseases including cataract, deafness, oculodentodigital dysplasia, keratitis-ichthyosis-deafness syndrome, Charcot-Marie-Tooth neuropathy, and leukodystrophy (*8*).

GJPs act as coordinated and dynamic platforms that enhance intercellular signaling. Their dynamics are influenced by a range of factors, including posttranslational modifications, lipid composition, and interactions with regulatory proteins (*9*–*16*). Moreover, GJPs, particularly those formed by connexin 43 (Cx43) have been shown to interface with cytoskeletal networks and cytoskeleton-associated proteins such as zonula occludens-1 (ZO-1) via the C-terminal domain (CTD) (*17*, *18*). These interactions stabilize and modulate the size of GJPs (*19*). The deletion of the Cx43 CTD results in the absence of GJPs, highlighting its significance for assembly (*20*). The impairment of these regulatory mechanisms has important implications for tissue physiology and disease. In cardiac arrhythmias, changes in GJP stability triggered by oxidative stress or altered the connexin composition can disrupt electrical impulse conduction (*21*). In cancer, the dysregulation of GJPs has been linked to tumor progression (*22*).

Current understanding of gap junction plaque structures has largely been informed by electron microscopy of immunogold-labeled freeze-fracture samples, immunocytochemistry, and studies using fluorescently tagged connexins, all of which have aimed to elucidate the structural organization, assembly mechanisms, and function of GJCs within plaques (*23*). In addition, atomic force microscopy has revealed conformational changes in purified connexin 26 plaques, particularly in the cytoplasmic domains and extracellular surfaces (*24*). Despite these efforts, the molecular-level organization of GJPs and the specific interactions that drive their assembly remains poorly understood. More recently, cryo–electron microscopy (cryo-EM) has provided a wealth of structural details on individual connexin channels shedding light on the effects of pathogenic mutations (*25*–*27*), as well as pore dynamics (*28*, *29*), pH-dependent gating (*30*), and voltage-dependent gating (*4*). However, this reductionist approach inherently omits the complexity of a native plaque environment. Notably, the CTDs have remained unresolved in these structures.

Here, we aimed to elucidate the structural architecture of the gap junction plaque in situ, focusing on Cx43 in human cells. To this end, we used cryo–electron tomography (cryo-ET) in combination with cryo–focused ion beam scanning electron microscopy (cryo-FIB/SEM) to visualize GJPs in their native cellular context. This approach enabled us to resolve molecular-level structural features of the GJC lattice in a near-native state. To gain further insight into the molecular interactions that underpin GJP formation and stability, we complemented our structural analysis with coarse grained (CG) molecular dynamics (MD) simulations. Our in situ structure of the connexin dodecamer in an open state, resolved at 14-Å resolution, revealed a key role for the CTDs in mediating lateral channel-channel contacts and provides a blueprint for understanding the molecular mechanisms that govern gap junction plaque assembly.

## RESULTS AND DISCUSSION

### Targeting gap junctions from micropatterned human cells for cryo-ET

To determine the ultrastructure of the gap junction plaque by cryo-ET, we established a workflow for preparing lamellae of cell-cell contacts by cryo-FIB/SEM (*31*). We generated a human embryonic kidney (HEK) 293T cell line stably expressing Cx43 tagged with enhanced green fluorescent protein (eGFP). Previously, it has been shown that C-terminally GFP-tagged connexins cluster normally to GJPs (*32*). This tagging, however, has been shown to increase the GJP size in HeLa cells (*33*). While advantageous for structural studies, this suggests that GFP fusion may interfere with interactions to regulatory factors or the dynamics of GJP assembly or disassembly.

The HEK293T-Cx43-eGFP cells were grown on micropatterned cryo-EM grids (fig. S1; see Materials and Methods). These grids enabled the enrichment of cells at the center of the grid squares and prevented cell overcrowding, thereby improving the throughput of lamella preparation (*34*). Of the 14 different patterns tested, an hourglass-shaped pattern proved advantageous for positioning GFP-positive cell-cell contacts in the middle of the grid squares. Cells on the micropatterned grids were subjected to fluorescence-targeted milling of 200-nm-thick lamella by cryo-FIB (fig. S2). Correlating the GFP signal from cryo–fluorescence light microscopy (cryo-FLM) with low-magnification cryo-EM images confirmed the presence of cell-cell contacts in lamella. The GFP-positive cell-cell contacts were 3.4 ± 1.4 μm (*n* = 5) long. The GFP signal was interrupted by 0.4 ± 0.2 μm (*n* = 7) long gaps. A closer examination of the cryo-EM images revealed that in areas devoid of GFP signal, the cell-cell contact was absent, and the two membranes were detached and curved away from each other forming a cavity (fig. S2F). These results show that HEK293T-Cx43-eGFP cells are suitable for targeting human GJPs by correlative imaging.

### Cryo-ET of the human gap junction plaque

The Cryo-EM of 19 lamellae milled from three cryo-EM grids of adherent HEK293T-Cx43-eGFP cells revealed the presence of cubic ice, possibly due to the relatively large thickness of the cells in the areas of cell-cell contacts, resulting in incomplete vitrification (fig. S2G). To overcome this for cryo-ET, we prepared grids in 10% glycerol as a cryo-protectant (*35*). A total of 20 lamellae milled from such a grid showed no signs of cubic ice, suggesting that they were suitable for structure determination by subtomogram averaging. The continuous GFP signals were shorter in the presence of 10% glycerol (0.5 ± 0.4 μm; *n* = 31) than in its absence (1.5 ± 0.9 μm; *n* = 13). A closer examination of the images revealed multiple punctate signals in the cytoplasmic regions. These signals likely correspond to connexosomes (annular gap junctions), vesicular structures formed by the endocytosis of GJPs (*36*). Their internalization may have been induced by osmotic effects of 10% glycerol and left a vacuole-like discontinuity at the plasma membrane devoid of connexins and thus lacking GFP signal (fig. S2I).

We used cryo-FLM for targeting 112 tilt series from the 20 lamella and reconstructed cryo-ET tomograms from 26 tilt series of suitable quality with a cell-cell contact region. The cell-cell contacts in these tomograms revealed repeated densities bridging the plasma membranes from the opposing cells (Fig. 1, B and C). In orthogonal view directions, these densities were seen to be ordered in a hexagonal lattice, reminiscent of the models of locally ordered connexin lattices (Fig. 1D) (*37*). As these structures are positive for the GFP signal, we attribute them to GJPs. The visual inspection of the tomograms revealed, in addition, organelles in proximity to the GJPs, such as a mitochondrion (Fig. 1B) and a Golgi apparatus (fig. S4A). Furthermore, vesicles, possibly involved in trafficking, were seen in proximity to microtubules and GJPs (fig. S4, B and C). In one instance, a coated vesicle, possibly in the process of membrane fusion, was connected to the plasma membrane next to a GJP (fig. S4D). We also observed a connexosome (400 nm in diameter) in the process of being internalized by endocytosis (fig. S4, E and F). These observations suggest that the HEK293T-Cx43-eGFP cells supplemented with 10% glycerol are a suitable model system for studying the cellular architecture in the context of human GJPs.

To map the molecular arrangement of the gap junctions, we used template matching to detect the positions of Cx43 GJCs, in addition to 80*S* ribosomes and microtubules, in 26 tomograms (see Materials and Methods; Fig. 1E). To account for possible effects of 10% glycerol on structure determination from these samples, as a control, we determined the structure of the 80*S* ribosome to 10-Å resolution from 6135 particles (fig. S3). This and an earlier 4.9-Å average of 80*S* ribosomes calculated using a significantly larger dataset (15,628 particles) from HEK293 cells with 10% glycerol (*35*) show that samples prepared this way are suitable for subtomogram averaging. We further quantified the distances of ribosomes, in addition to microtubules, to the GJCs by calculating their closest pair-wise Euclidean distances (Fig. 1F). Ribosomes were apparently excluded from the vicinity of the GJPs within a 22-nm-wide ribosome exclusion zone (REZ). This suggests that the relatively large Cx43 CTDs and possibly other proteins interacting with this domain, in addition to actin filaments (*13*), occupy this region. Microtubules were occasionally present within REZ (Fig. 1F), consistent with their reported interaction with Cx43 (*17*, *38*).

### Curvature and asymmetry in the GJC lattice

**Fig. 2.**
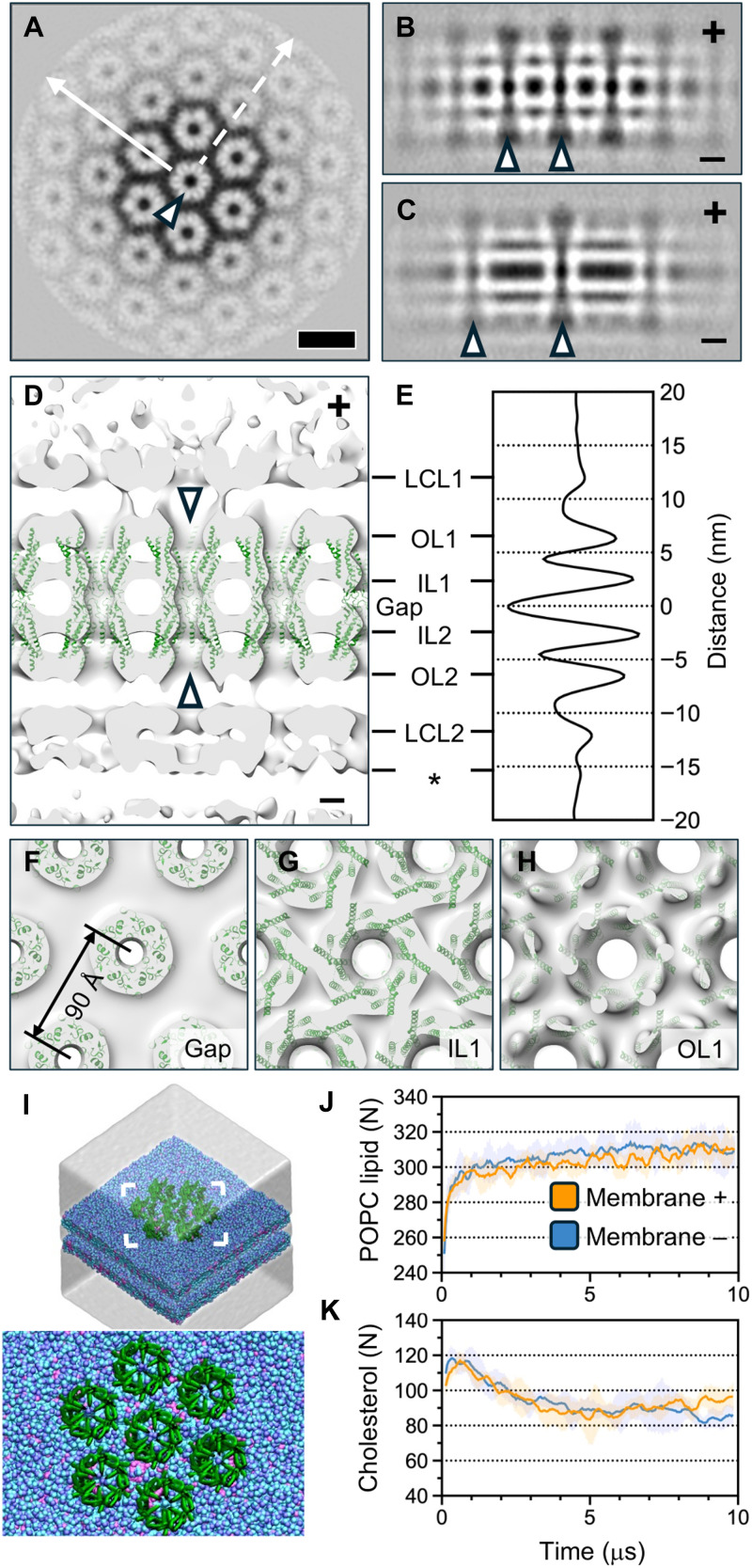
Subtomogram averaging of Cx43 GJCs. (**A**) Slice through a subtomogram average of the Cx43 lattice is shown. One channel is indicated (arrowhead). Scale bar, 10 nm for (A) to (C). (**B**) Slice through the subtomogram average, along the solid line in (A). (**C**) Slice along the dashed line in (A). Channels are indicated in (B) and (C) (arrowheads). (**D**) Isosurface of a subtomogram average. A low-pass filter to 20-Å resolution has been applied to improve the interpretability of the lateral layers. Cx43 atomic models (PDB: 7Z22, residues 17 to 105 and 151 to 235) are shown in green. Two hemichannels forming a full channel are indicated (arrowheads). The convex (+) and concave (−) sides of the lattice are indicated in (B) to (D). (**E**) Density distribution as a function of distance from the lattice midpoint is shown. Density is in arbitrary units. The inner (IL) and outer (OL) leaflets of the two lipid bilayers are marked. The regions corresponding to intracellular density bridging Cx43 hexamers, denoted as lateral-contact layers (LCLs), are labeled. An additional layer of density on the concave side of the gap junction is indicated with an asterisk. (**F** to **H**) Isosurface renderings are shown from the top (+ to – direction) for the extracellular region (gap), inner leaflet (IL1), and intracellular densities (OL1). (**I**) Rendering of the CG MD simulation setup. The connexins are green, the POPC lipids are blue (head groups in a darker shade), and cholesterol is magenta. The solvent water is rendered as a transparent cube. The inset shows the area indicated. (**J** and **K**) Number of POPC lipids (J) and cholesterol molecules (K) around the centermost gap junction hemichannel is plotted as a function of the simulation time for both the membranes (+ and – sides).

To determine the ultrastructure of the gap junction in situ, we used subtomogram averaging. The classification of 30,338 subtomograms of GJC lattice patches from 26 cryo-ET tomograms revealed lattices with varying degrees of cylindrical and saddle curvatures (fig. S5, A to C). The direction of the connexin lattice was observed to be uncoupled from the principal axes of curvature. Conversely, connexin patches with the same curvature and lattice orientation clustered in the same local areas of the GJP (fig. S5, D to F). We cannot, however, exclude the possibility that the 10% glycerol used as a cryo-protectant affects membrane curvature, possibly through osmotic effects, or the gap junction structure itself. We extracted, aligned, and averaged 11,904 subtomograms with a clear GJC channel lattice to reconstruct a local patch of the GJCs at 14-Å resolution (Fig. 2 and fig. S3). The volume, reconstructed with C6 symmetry, reveals a lattice of Cx43 channels (Fig. 2, A to C). Each channel is formed by two opposing Cx43 hexamers as has been reported earlier (Fig. 2D) (*39*). We quantified the density distribution in the model, considering the radius of curvature (1.05 μm) of the GJC lattice in this reconstruction. An additional layer of density was observed on the concave side, but not the convex side, of the GJC lattice, possibly corresponding to other proteins inducing or sensing the negative curvature (Fig. 2, D and E). The total thickness of the Cx43 GJP measures ~30 nm. The channel-to-channel distance, measured from the channel centers, is 90 Å, consistent with a previous report (Fig. 2F) (*40*).

### Intracellular ordered structural elements bridge GJCs

To understand channel-channel interactions in the lattice, we fitted an atomic model of the Cx43 channel (*25*) into the density, with a cross-correlation coefficient of 0.59 both for the central dodecamer and its neighbor, which were fitted independently. This fitting established both the positions and the in-plane orientations of the GJCs in the lattice (Fig. 2D). Fitting of this GJC model, which encompasses the transmembrane (TM) regions in addition to the extracellular loops (covering ~50% of the entire structure; see Fig. 1A), revealed no contacts between individual GJCs (Fig. 2, F to H). Instead, CG MD simulations of connexin lattices in membranes containing 1-palmitoyl-2-oleoyl-*sn*-glycero-3-phosphocholine (POPC) and cholesterol showed that, on average, ~16 cholesterol and 50 POPC molecules reside between any two connexin hemichannels (Fig. 2, I to K). In comparison, additional simulations with more complex plasmamembrane-like lipid mixtures showed ~13 cholesterol molecules in addition to a lipid belt between hemichannels (fig. S6). A corollary of this finding is that the lateral channel-channel contacts needed to assemble an ordered Cx43 GJC lattice must reside in the intracellular region encompassing the intracellular loop (IL; residues 105 to 150) and the CTD (residues 236 to 382). Consistent with this notion, ordered density resides proximal to both membranes (Fig. 2D). We assign this density to the Cx43 CTDs and the ILs. We cannot, however, exclude the possibility that the C-terminal eGFP-tag or connexin-interacting proteins also contribute to this density. Notably, the CTD is disordered in the Cx43 GJC structure which has been determined earlier using purified channels (*25*). Our data suggest that it becomes at least partially ordered in the cellular context and that it interacts with additional cellular factors, especially on the concave side of the slightly curved membrane.

Our Cx43 GJC lattice model facilitates the comparison to other modeled connexin lattices (fig. S7). Cx26 is one of the smallest connexins (226 residues) and largely lacks the CTD. It has been reported to have much closer channel-to-channel distances (77 ± 5 Å) (*24*) compared to Cx43 (90 Å). Cx26 GJC lattice model suggest that the channel-channel contacts are created by the short C-terminal tail (222 to 227) possibly in addition to the TM helix 4 (218 to 221). This difference suggests that Cx43 has evolved a looser lattice where contacts are mediated by its extended CTDs, possibly to allow for a finer regulation of the GJP.

### In situ structure of the human GJC

**Fig. 3.**
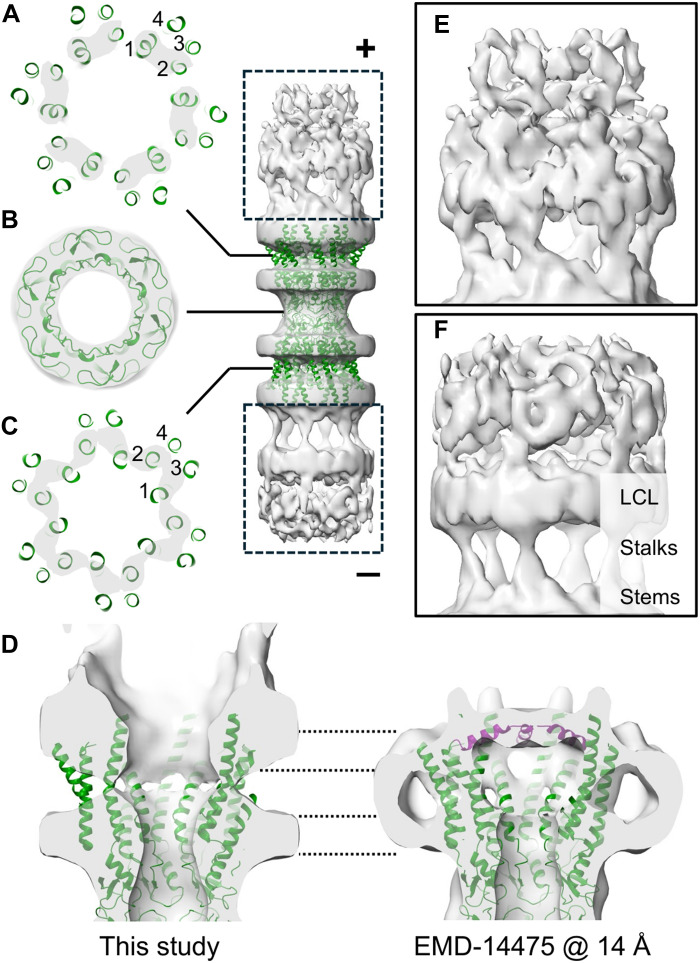
Structure of the Cx43 channel. (**A** to **C**) Isosurface renderings of the cryo-ET subtomogram average at 14-Å resolution segmented with a cylindrical mask are shown as gray transparent surfaces, together with a partial atomic model of the Cx43 channel (PDB: 7Z22, residues 17 to 105 and 151 to 235). The inset indicates the level of the cross sections taken from the middle of the top bilayer (A), the middle of the extracellular loops (B), and the middle of the bottom bilayer (C). The TM helices are numbered 1 to 4 for one Cx monomer in both hemichannels. The convex (+) and concave (−) sides of the gap junction are indicated. (**D**) Close-up of the hemichannel cryo-ET map (this study) and a single-particle cryo-EM structure of the Cx43 hemichannel (EMD-14475), low-pass filtered to the same resolution (14 Å) for comparison. At this resolution, the N-terminal helices (residues 1 to 16, purple) closing the channel are visible in the cryo-EM density. The dashed lines indicate the position of the two membrane leaflets in both structures. (**E** and **F**) Close-ups of the intracellular areas indicated in the inset (dashed rectangles) are shown. In both, a stalk-like density can be seen, connecting the density below it (stem) and the density above it [lateral contacts layer (LCL)].

The in situ structure of the GJC shows a varying degree of order (Fig. 3). The extracellular loops connecting the two hemichannels, in addition to the TM helices 1 and 2, are relatively well resolved. In contrast, TM helices 3 and 4 are unresolved, suggesting relatively higher mobility, structural variation, or deviation from a strict sixfold symmetry in these helices that are distal from the channel pore (Fig. 3, A to C). To determine whether the pore observed in situ is open or closed, we compared its cryo-ET map to the previously published single-particle cryo-EM structure of Cx43 GJCs in nanodiscs (Fig. 3D) (*25*). We first low-pass filtered this map to 14-Å resolution to facilitate comparison to our cryo-ET map at this resolution. In this structure, the N-terminal helices (residues 1 to 16) form a lid that closes the channel, and this feature is resolved at 14-Å resolution. As this lid feature is absent in our in situ structure, we conclude that the channels are predominantly in an open conformation in our in situ data. Because of the averaging approach applied, we cannot, however, exclude the possibility that a fraction of the channels is in a closed or an intermediate conformation.

Both intracellular regions proximal to the membranes show partially ordered density (Fig. 3, E and F). In both regions, a stalk-like density is seen protruding from a relatively bulkier region termed “stem,” and connecting to a layer of density termed “lateral contacts layer.” This layer of density most likely corresponds to the observed channel-channel contacts (see Fig. 2D; CTD1 and CTD2), which are most likely created by the CTDs due to their extensive size (residues 236 to 382). Fitting of a predicted full-length Cx43 GJC structure places the IL in the stem region of the intracellular density (fig. S8). Furthermore, a putative helix-loop-helix (HLH) motif (R299 to E352) is predicted within the CTD (fig. S8). Nuclear magnetic resonance (NMR) experiments of the CTDs have shown that in human Cx43 S314 to I327 and Q342 to A348 exhibit α-helical structure (*41*). Furthermore, an HLH motif (A315 to A348) has been detected in rat Cx43 CTD (*42*), which has a high degree of sequence identity to that of human Cx43 (95%). We hypothesized that this motif may create either dimeric or trimeric channel-channel contacts. Furthermore, NMR experiments have shown that three regions within this motif (R299 to Q304, S314 to I327, and Q342 to A348), in addition to one preceding it (M281 to N295), have the tendency to drive Cx43 CTD dimerization at low pH (*43*). We propose that the residues before the HLH (236 to 314) contribute to the relatively ordered stem and stalk regions observed in our cryo-ET structure. Last, the superposition of the predicted Cx43 GJC full-length model on our GJC lattice revealed a putative network of lateral interactions between HLHs motifs (fig. S8E). Together, these observations suggest that the Cx43 CTD creates the channel-channel interactions required for the GJP assembly via the dimerization of the putative HLH motifs. Consistent with this hypothesis, the fluorescence imaging of a Cx43 HLH-deletion mutant showed that the number of GJPs was significantly reduced when compared to the full-length Cx43 (fig. S8, F and G). However, the localization and the experimentally determined structure of the CTD require higher-resolution cryo-ET, possibly using high-pressure freezing in the absence of cryo-protectant (*44*).

To conclude, by establishing a cryo-ET workflow in HEK293T cells expressing connexin 43, we enabled the direct visualization of connexin lattices within human GJPs in situ. Cryo-protection with glycerol preserved the ultrastructure of cell-cell contacts and allowed high-resolution imaging of GJPs. These plaques adopt a semi-ordered lattice of GJCs embedded in a variably curved membrane. The lattice exhibits further structural asymmetry marked by additional density on the concave cytoplasmic face, potentially corresponding to binding partners or curvature-sensing components. As individual connexin 43 channels are not in direct contact but instead spaced by lipids and cholesterol, we propose that lateral channel-channel contacts are mediated by CTDs. These domains, disordered or absent in previous studies of detergent-purified channels, are ordered in situ and appear to be essential for lattice gap junction assembly. While the precise conformation and positioning of the CTD remain unresolved at this resolution, our data strongly support its role as the primary mediator of lateral channel organization in situ. Higher resolution will be required to resolve their conformations and molecular contacts in detail. Furthermore, the physiological significance of the HLH motif needs to be established in further studies. Together with the recent in situ structure of innexin-based gap junctions in *Caenorhabditis elegans* (*45*), our work demonstrates that cryo-ET can now resolve native gap junction organization. In conclusion, our study provides a framework for understanding the spatial organization of human gap junctions in the cellular context.

## MATERIALS AND METHODS

### Connexin 43 cloning and stable cell line generation in HEK293T

Human connexin 43 (Uniprot ID P17302) plasmid was obtained from the Human ORFeome Library (Genome Biology Unit, Helsinki Institute of Life Science, University of Helsinki). Connexin 43 with a C-terminal 3C-eGFP-SpyTag003 (*46*) tag was cloned into pURD vector (*47*) and used for stable cell line generation in HEK293T cells. HEK293T cells were cultured in Dulbecco’s modified Eagle’s medium (DMEM) supplemented with 10% fetal bovine serum (FBS; Gibco), 5% nonessential amino acids (NEAA; Gibco), and 5% l-glutamine (Gibco) and incubated at 37°C in a 5% CO_2_-containing atmosphere. To obtain HEK293T cells stably expressing Cx43-3C-eGFP, cells were transfected with 1 μg of pURD vector harboring Cx43-3C-eGFP and 3 μg of integrase expression vector (pgk-φC31/pCB92) using Lipofectamine 2000 (Thermo Fisher Scientific). Confluent cells were selected using puromycin (4 μg/ml; Thermo Fisher Scientific) for 2 weeks. For clonal line generation, GFP-positive cells were isolated by fluorescence-activated cell sorter (FACS) instrument (FACSAria II, BD Biosciences). Clones were monitored and expanded, and the fluorescence intensity was validated using a plate reader.

### Micropatterning of cryo-EM grids

Cryo-EM grids (Quantifoil R2/2 Au 200 mesh with a SiO_2_ foil) were micropatterned using a micropatterning system (Alvéole PRIMO). The grids were plasma cleaned at 0.8 torr for 50 s using a tabletop high-power expanded plasma cleaner (Harrick Plasma PDC-002). For micropatterning the grids, a two-step passivation approach was used. First, the grids were transferred onto a glass coverslip covered with parafilm, and 20 μl of 0.01% of poly-l-lysine solution (CAS number 25988-63-0, P4707, Sigma-Aldrich) was immediately added. The glass coverslip containing the grids was transferred into a humidified chamber and incubated overnight at 4°C. Next, the grids were washed three times in 0.1 M Hepes (pH 8.5) and incubated in 20 μl of methoxypolyethylene glycol (mPEG)–succinimidyl valerate solution [50 mg/ml in 0.1 M Hepes (pH 8.5), molecular weight 5000, mPEG-SVA, Laysan Bio) for 1 hour at room temperature. The grids were washed three times in deionized water. Excess water from each grid was removed by blotting, and the grids were air-dried. A 3-μl aliquot of a 1:3 dilution solution of photoinitiator (PLPP gel, Nanoscalelabs) in 70% ethanol was added to the surface of each grid and incubated in the dark until the gel was completely dry. The grids, fixed in position on a glass coverslip by polydimethylsiloxane stencil with the SiO_2_ foil side up were placed on an inverted widefield microscope (Nikon Eclipse Ti-E) mounted with Alvéole PRIMO. Each grid was then exposed to ultraviolet (UV) light at 100 mJ/mm^2^. After micropatterning, the grids were washed three times in deionized water and further washed three times in 1× phosphate-buffered saline (PBS). The grids were kept in a 35-mm dish, hydrated in 1× PBS, and stored at 4°C until they were ready to be used.

### HEK293T cell culture and seeding

HEK293T cells stably expressing Cx43-3C-eGFP were maintained in DMEM with 10% FBS, 5% NEAA, and 5% l-glutamine and incubated at 37°C in a 5% CO_2_-containing atmosphere. The micropatterned grids were sterilized in 70% ethanol for 5 min under UV light and subsequently washed three times in deionized water and three times in 1× PBS. To functionalize the micropatterned grids, 20 μl of extracellular matrix (ECM) solution containing 15 μl of fibronectin (catalog number 1918-FN-02 M, Bio-Techne) mixed with 5 μl of fibrinogen-647 (fibrinogen from human plasma, Alexa Fluor 647 conjugate, Life Technologies Corporation) at 3:1 ratio in 1× PBS was added to each grid and incubated at room temperature for 1 hour. The ECM solution was removed from the grids by using pipette, and the grids were washed three times in 1× PBS. The grids were conditioned in DMEM by washing three times. HEK29T cells were trypsinized, resuspended in DMEM, and filtered through a 40-μm cell strainer. A 5-μl aliquot of cell suspension at a concentration of 5 × 10^4^ cells/ml was added to each grid in drops sequentially until a minimum of two to three cells per grid square was achieved. After 2 hours, grids were washed with DMEM and incubated at 37°C in 5% CO_2_-containing atmosphere for ~24 hours before vitrification.

### Cryo-FIB/SEM lamella preparation

The cells were vitrified into liquid ethane using a manual plunge freezer. Optionally, 5 μl of 10% glycerol diluted in 1× PBS was added to the cells immediately before plunge freezing. The cryo-FIB of frozen-hydrated HEK293T cells was performed using an Aquilos2 dual-beam FIB-SEM microscope (Thermo Fisher Scientific). Metallic platinum was deposited on the grids (1 kV, 10 mA, 10 Pa, 15 to 20 s), followed by organic platinum deposition via a gas injection system for 10 to 20 s. AutoTEM-cryo software (Thermo Fisher Scientific) was used to mill the cells with a milling angle of 12° to a thickness of 1 μm in three steps with decreasing ion beam currents (1, 0.5, and 0.3 nA) at 30 keV. Lamella polishing was achieved by thinning to a final thickness of 200 to 300 nm at 50 pA. To complete the milling process, the lamellae were sputter-coated with platinum (1 kV, 10 mA, 10 Pa, 5 to 15 s). To prevent the lamella from breaking or bending, microexpansion joints were used during milling.

### Cryo-FLM imaging of the lamellae

The milled cryo-EM grids were imaged using a super-resolution confocal microscope (Zeiss LSM 900 Airyscan) equipped with a cryo-stage (Linkam). A low-magnification air objective (5×) in wide-field mode was used to acquire an overview image of the lamellae. This was followed by acquiring z-stacks with a 0.5-μm spacing covering 4 to 6 μm in Airyscan mode with 488 laser line and a 100×/0.75 numerical aperture (NA) air objective (Zeiss Plan-Neofluar) using a pixel size of 79 nm. Reflection-mode images were acquired for each *z*-plane. To increase the signal-to-noise ratio of each image stack, the stacks were averaged, and maximum intensity projections were generated. The correlation of acquired Airyscan images and low-magnification transmission electron microscopy images (lamella maps) was performed with Icy eC-CLEM (*48*) and Fiji BigWarp (*49*) plugins by using the lamellae shape and features visible in the reflection images as landmarks.

### Cryo-ET data acquisition

Cryo-ET data were collected on a Titan Krios G4 transmission electron microscope operated at 300 kV and equipped with a Falcon4i direct electron detector and a SelectrisX energy filter (Thermo Fisher Scientific). The lamella pretilt axis was aligned with the microscope stage tilt axis. Data were acquired using SerialEM software (v 4.1.0beta) (*50*) and PACE-tomo (*51*). To record 112 tilt series from 20 lamella, a dose-symmetric tilting scheme (*52*) from −40° to +64° with 2° increments was used, starting from the lamella pretilt angle (12°). Tilt series were acquired in counting mode at 3 to 6 μm under focus using a pixel size of 2.4 Å/pixel. For each tilt, an electron exposure of 3 e^−^/Å^2^ per tilt was used, resulting in a total electron exposure of ~160 e^−^/Å^2^.

### Tomogram reconstruction and subtomogram averaging

Cryo-ET data were processed using the Warp-RELION-M pipeline (*53*). A subset of 26 tilt series was selected from the total of 112 by visual inspection for further processing. After motion correction and contrast transfer function (CTF) estimation of the frame series in WarpTools (v2.0.0), tilt images were filtered by defocus and CTF resolution, retaining images with a defocus higher than 2.6 μm and a resolution of 11 Å or better. Down-sampled tomograms (9.6 Å/pixel) were reconstructed for template matching in PyTom Python package pytom-match-pick (*54*). An initial template volume of the connexin lattice was generated by tracing the gap junction regions manually in Dynamo (v.1.1.532) (*55*) and averaging the patches with D6 symmetry in RELION (v5.0beta) (*56*). For template matching of the 80*S* ribosome and microtubules, EMD-15815 and EMD-6351 were used, respectively. The templates were low-pass filtered to 40-Å resolution. The coordinates were extracted using a top-hat filter (*54*). The coordinates for connexin patches and microtubule segments were manually curated in UCSF ChimeraX (*57*) using ArtiaX plugin (*58*). For connexin patches, only the coordinates corresponding to a 2D surface matching the gap junction region in the tomograms were retained. For microtubule segments, only the coordinates corresponding to 1D tracks of the filaments were retained. For connexins, the tilt and psi Euler angles were added as priors using a custom Python script. The connexin and ribosome particles were exported to RELION using Warp at 4.8 Å/pixel sampling and a box size of 128 pixels to classify and refine the particles. For connexins, C2 symmetry was applied to retain different curvatures of the connexin patch. The resulting averages were used for the second round of template matching in pytom-match-pick and manual curation in ArtiaX. Further classification in RELION revealed two types of curvature in the connexin patches. These two templates were used for the third and final round of template matching in PyTom. The coordinates were consolidated from two separate template matching runs, keeping the highest correlating match for each position using a custom Python script. After refinement of all datasets in RELION, the connexin, ribosome, and microtubule particles were exported to M for multiparticle refinement (*53*). The particles were refined in seven stages: (i) Five rounds of 1 × 1 image warp with particle refinement. (ii) Five rounds of 4 × 4 image warp with particle refinement. (iii) Per-tomo weighting followed by five rounds of particle refinement. (iv) Per-tomo weighting followed by five rounds of particle refinement. (v) Exhaustive defocus parameter estimation followed by five rounds of 4 × 4 image warp with particle refinement. (vi) Per-tomo weighting followed by five rounds of particle refinement. (vii) Five rounds of 4 × 4 × 1 × 10 volume warp with particle refinement. The refined connexins and ribosome particles were exported to RELION at 2.4 Å/pixel sampling and using a box size of 256 pixels for a final round of refinement. C6 symmetry was imposed on the connexin map. The Fourier shell correlation (threshold 0.143) was used to estimate both the global and local resolutions from two independent half datasets in RELION. The connexin map was sharpened using an ad hoc inverse B factor of −500 Å^2^. The cryo-EM maps, including that of the microtubule segment at 40-Å resolution, were plotted back on the tomograms in ChimeraX using ArtiaX plugin. The ribosome-to-connexin and microtubule-to-connexin distances were calculated using a custom Python script. The density distribution of the gap junction was calculated from a cryo-EM reconstruction low-pass filtered to 20-Å resolution within an aperture of 15 nm and considering the radius of curvature 1.05 μm using a custom Python script. The radius and directions of curvature were determined by fitting a surface (a sphere, a cylinder or a saddle) to the center-of-mass (COM) of Cx43 models rigid body fitted to the 3D class averages (with C2 symmetry) or the final connexin map (with C6 symmetry) using a custom Python script.

### Molecular modeling and CG MD simulations

To probe the dynamical behavior of lipids and their interactions with Cx43 GJCs, we created a CG model based on the 14-Å cryo-ET structure of Cx43 GJC lattice with martinize2 software (version 2.9) and using the Martini 3 forcefield (*59*). The residues corresponding to the intracellular loop (106 to 150) were modeled with AlphaFold2 (*60*). The double membrane CG model of the protein complex comprises seven Cx43 hemichannels immersed in each of the two POPC/cholesterol containing phospholipid bilayers. The phospholipid bilayer consisted of POPC and cholesterol in a 70:30 ratio or POPC, cholesterol, POPE, and sphingomyelin in a 30:30:20:20 ratio. The CG protein-membrane system was solvated with the standard Martini 3 water and ion beads with a 150 mM concentration of Na^+^ and Cl^−^ ions using the insane.py script (*61*).

All MD simulations were carried out using the GROMACS software (version 2022) (*62*). To avoid any steric clashes, the CG system was energy minimized for 5000 steps. The arrangement of the GJCs was restrained in production simulations by applying a harmonic force constant of 1000 kJ mol^−1^ nm^−2^ on the backbone CG beads. The velocity-rescaling thermostat (*63*) and c-rescaling barostat (*64*) were used to maintain the temperature at 300 K and pressure of 1 bar, respectively. The reaction field algorithm was used to treat the coulombic interactions using ε_r_ = 15 (*65*). The implementation of the Verlet cutoff scheme was used with a Lennard Jones cutoff of 1.1 nm (*66*). Three 10- to 12-μs CG MD simulations were performed using a time step of 20 fs.

To estimate the number of lipids located between adjacent connexin hemichannels, we performed analysis using MDAnalysis (*67*). First, the COM of the central hemichannel was selected, and the distance to the COM of its adjacent hemichannels in the same membrane was used to define a radial cutoff (8.5 nm). All lipids (either POPC or cholesterol) within this radial region were selected and counted along three simulation trajectories of 10 μs each. The total number of lipids within the defined cutoff was then divided by six to approximate the number of lipids, i.e., POPC and cholesterol molecules shared between each pair of adjacent hemichannels. This procedure was applied independently for each membrane bilayer.

We predicted the structure of the full-length structures of Cx26 and Cx43 with D-I-TASSER (*68*). Dodecamers were created by matchmaker command in ChimeraX using the atomic coordinates Protein Data Bank (PDB): 7QEQ and PDB: 7Z22, respectively, as templates. Phosphorylation sites were retrieved from PhosphoSitePlus (*69*) and mapped onto the structure in UCSF ChimeraX.

### Fluorescence microscopy of GJPs

HEK293T stable cell lines expressing wild-type Cx43 and a HLH motif deletion mutant (Cx43-dHLH). Both constructs were tagged with SpyTag for fluorescence imaging. Cells were seeded onto coverslips precoated with poly-l-lysine at a density of 1 × 10^6^ cells/ml and incubated at 37°C in 5% CO_2_-containing atmosphere for 18 to 24 hours. Cells were fixed in 4% paraformaldehyde for 10 min at room temperature. Cells were washed in PBS and permeabilized in 0.1% Triton X-100 for 10 min at room temperature. The cells were washed in PBS and blocked in 3% bovine serum albumin (BSA) diluted in PBS at room temperature for 1 hour. The blocking buffer was discarded, and cells were incubated overnight in a cold room in fresh 3% BSA buffer containing 100 nM SpyCatcher protein conjugated to Alexa Fluor 647 NHS ester (Jena Biosciences). Cells were washed twice in PBS and mounted in FluoroMount G mounting medium with 4′,6-diamidino-2-phenylindole (DAPI). Confocal images were acquired on a fully motorized upright microscope (Leica SP8) with a 63× 1.35 NA CS2 glycerol immersion objective. Excitation wavelengths were set to 405 nm (DAPI) and 638 nm (Alexa Fluor 647), respectively. Images of both samples were acquired from random positions.

The quantitative and statistical analysis of GJPs per nucleus were performed by first counting the number of nuclei per image using the threshold function in Fiji software. In addition, the number of GJPs per image was estimated by visually inspecting and counting individual plaques formed between two or more cells. The GJP per nuclei ratios between the wild-type Cx43 and mutant Cx43-dHLH were compared using a two-tailed Mann-Whitney *U* test.
