# Supplementary material for: In situ structure of the human gap junction
Source: Sci Adv. 2026 May 13;12(20):eaea4183. doi: 10.1126/sciadv.aea4183 (PMC13170659; doi:10.1126/sciadv.aea4183)
Supplement: Supplementary file 1 — Figs. S1 to S8 [file sciadv.aea4183_sm.pdf]

Science Advances
AAAS

Supplementary Materials for

In situ structure of the human gap junction

Evans Eshriew *et al.*

Corresponding author: Juha T. Huiskonen, juha.huiskonen@helsinki.fi

Sci. Adv. **12**, eaea4183 (2026)
DOI: 10.1126/sciadv.aea4183

This PDF file includes:

Figs. S1 to S8

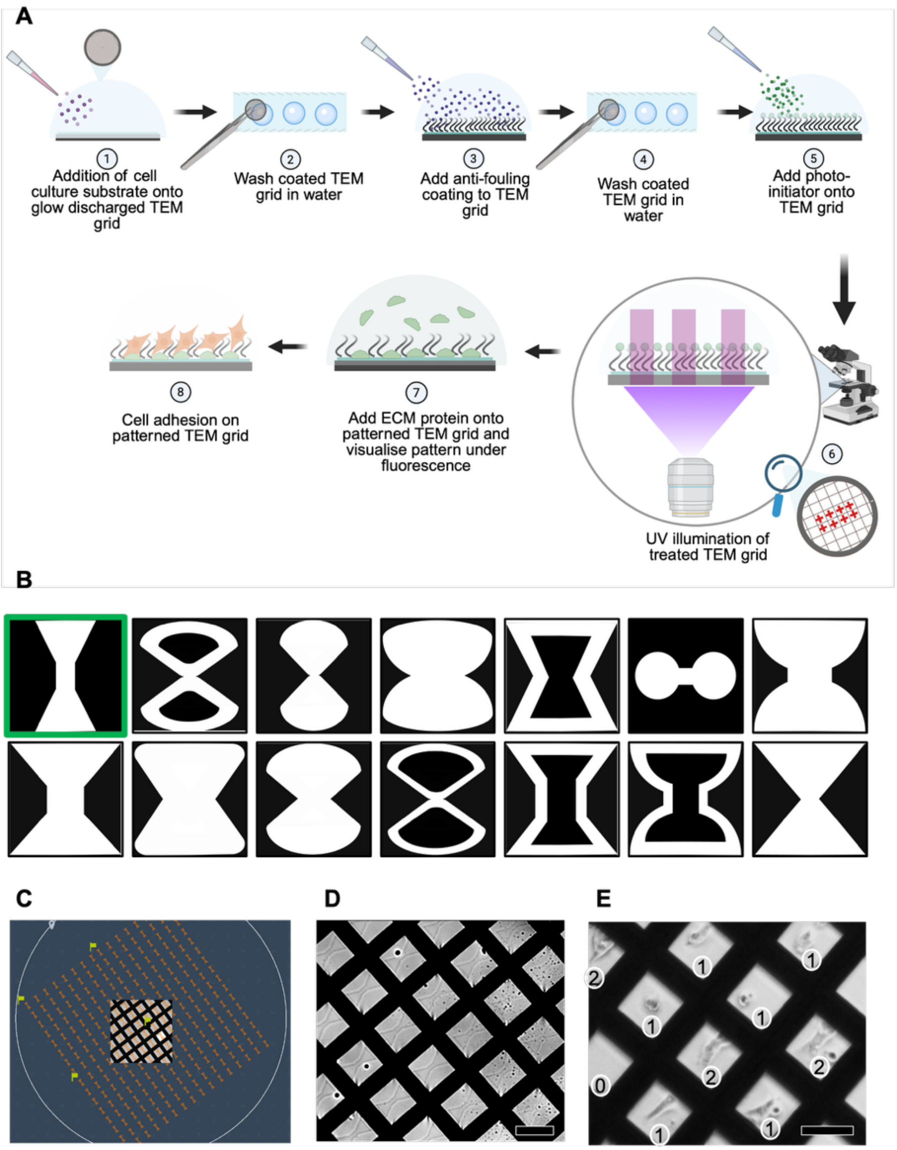

SFigure 1. Micropatterning of electron microscopy grids for targeting cell–cell junctions by cryogenic electron tomography. (**A**) A schematic representation of the micropatterning workflow. A specific pattern, placed over each grid square, is used to illuminate the grid with UV light. (**B**) Different patterns tested to guide cells to form a gap junction in the middle of the grid square. The selected pattern (a narrow hour-glass shape) is outlined in green. (**C**) A representative image of a cryo-EM grid overlaid with selected pattern. (**D**) A close-up of a cryo-EM grid after micropatterning shows hourglass-shaped pattern on each grid square. Scale bar, 100 µm. (**E**) HEK293T-Cx43-eGFP cells grown on micropatterned cryo-TEM grid. The numbers indicate the number of cells per each grid square. Scale bar, 100 µm.

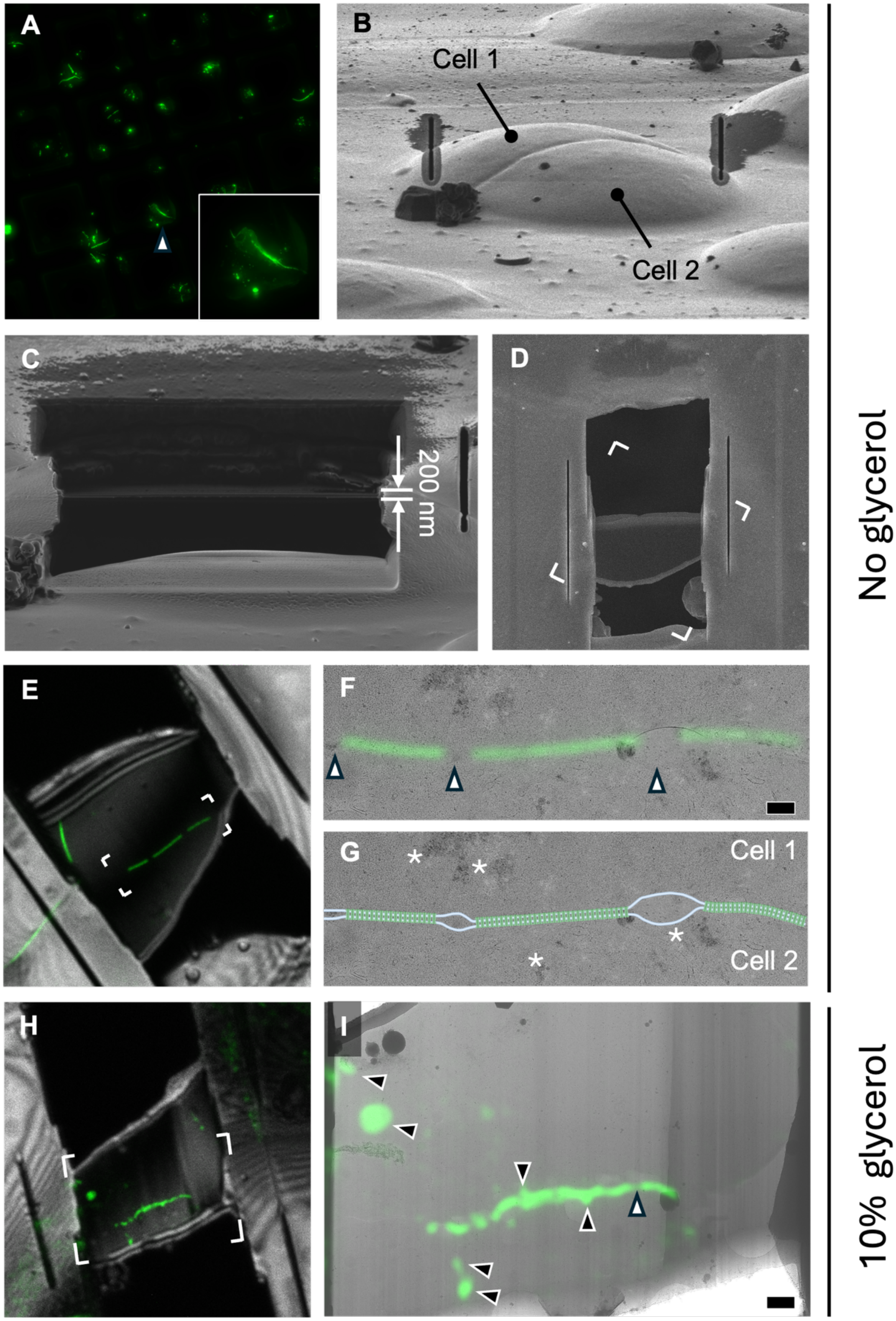

SFigure 2. Targeting gap junctions in HEK293-Cx43-eGFP cells by correlative cryogenic focused ion beam scanning electron microscopy (cryo-FIB/SEM). (**A**) Integrated fluorescence

light microscopy image of HEK293-Cx-eGFP cells without glycerol on a cryo-EM grid prior to cryo-FIB milling. The inset shows a feature marked with an arrowhead, corresponding to a gap junction plaque. (**B**) A scanning electron microscopy (SEM) image of two cells contacting each other. (**C**) The same area after milling a 200-nm-thick lamella using a focused ion beam (FIB). (**D**) The same lamella imaged from the top. (**E**) The area indicated in *d* imaged using cryo-fluorescence super-resolution light microscopy. The signal from eGFP-tagged connexin43 is in green. (**F**) The area indicated in *E* imaged using cryogenic transmission electron microscopy (cryo-TEM). The fluorescence signal from *e* is shown overlaid on the cryo-TEM image. Interruptions in the GFP signal are indicated with white arrowheads. Scale bar, 1 µm. (**G**) A schematic diagram is shown over the same area from *F* to illustrate the areas with Cx43 channels and cavities where this signal is absent. Areas with signs of non-amorphous ice have been indicated with asterisks. (**H**) A lamella of cells with 10% glycerol imaged using cryo-fluorescence super-resolution light microscopy. (**I**) The area indicated in *H* imaged using cryo-TEM. The fluorescence signal from *H* is shown overlaid on the cryo-TEM image. Some punctate GFP signals, possibly corresponding to connexosomes (annular gap junctions), are indicated with black arrowheads. Interruptions in the GFP signal are indicated with white arrowheads. Scale bar, 1 µm.

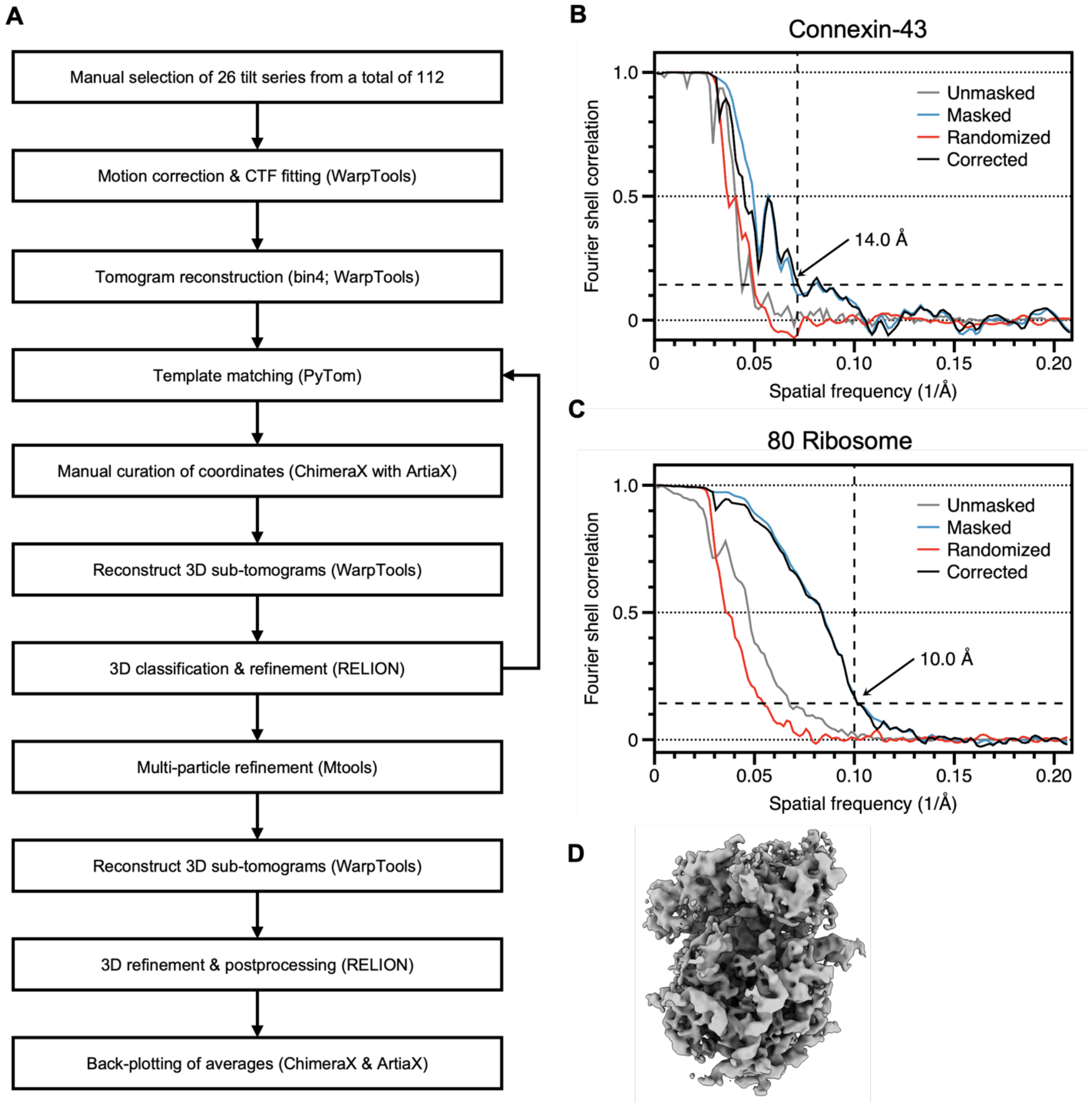

SFigure 3. Data processing workflow and resolution estimation. (**A**) Data processing workflow for the reconstruction of sub-tomogram averages. (**B**) Fourier shell correlation (FSC) of the connexin cryo-ET map. (**C–D**) FSC (*C*) and an isosurface rendering (*D*) of the 80S ribosome cryo-ET map.

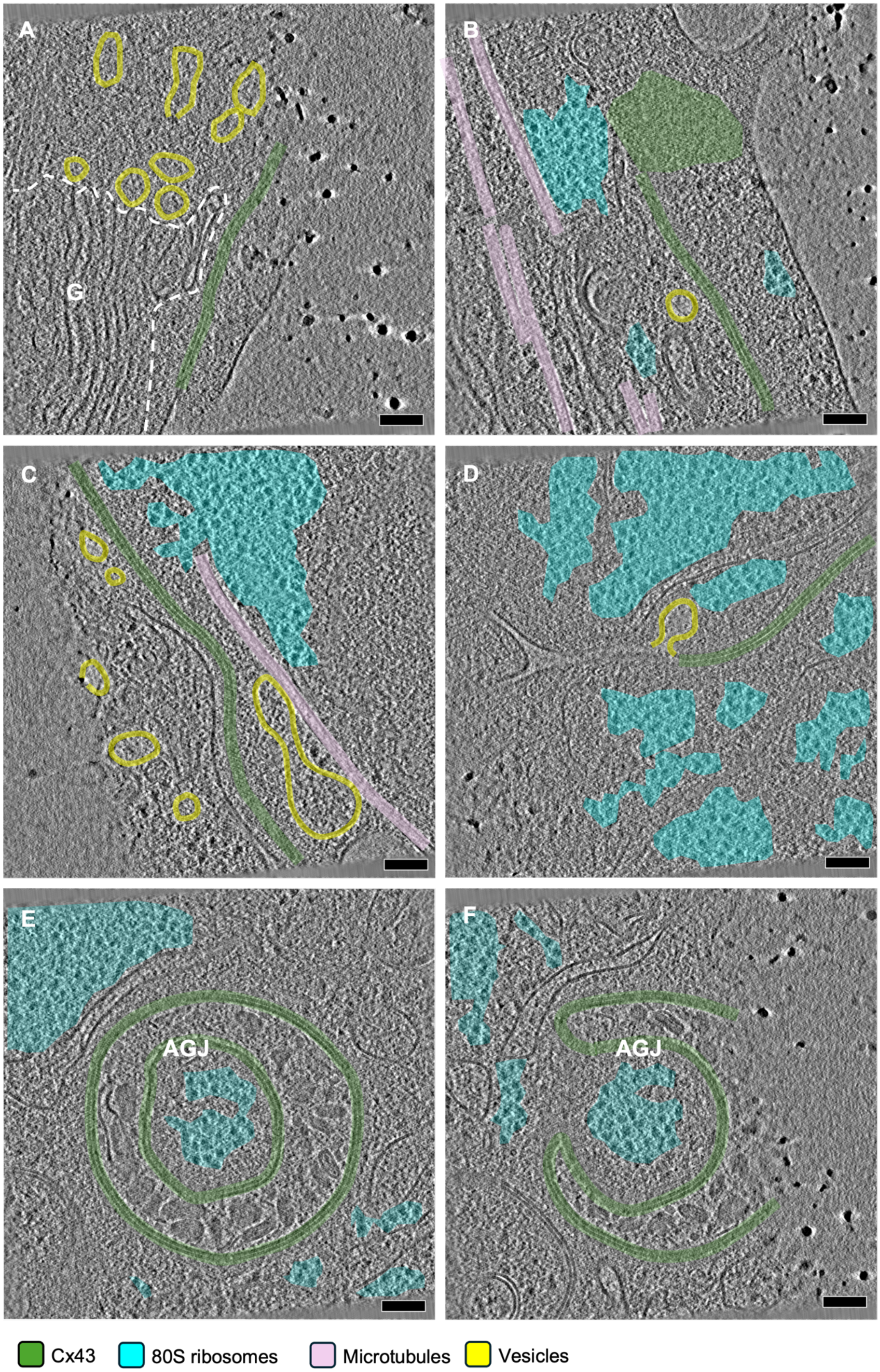
A
G
B
C
D
E
AGJ
F
AGJ
Cx43
80S ribosomes
Microtubules
Vesicles

SFigure 4. Cellular architecture in the vicinity of gap junctions. (**A–F**) 1-nm-thick slices through cryogenic electron tomography (cryo-ET) reconstructions of cell-cell junctions. Areas harbouring Cx43 channels (green), 80S ribosomes (cyan), microtubules (pink) and vesicles (yellow) have been indicated. A Golgi apparatus (G) has been labelled and outlined with a dashed line in *A*. A connexosome (annular gap junction, AGJ) in the process of being endocytosed is shown in two slices, separated by 80 nm in *E* and *F*. Scalebars, 100 nm.

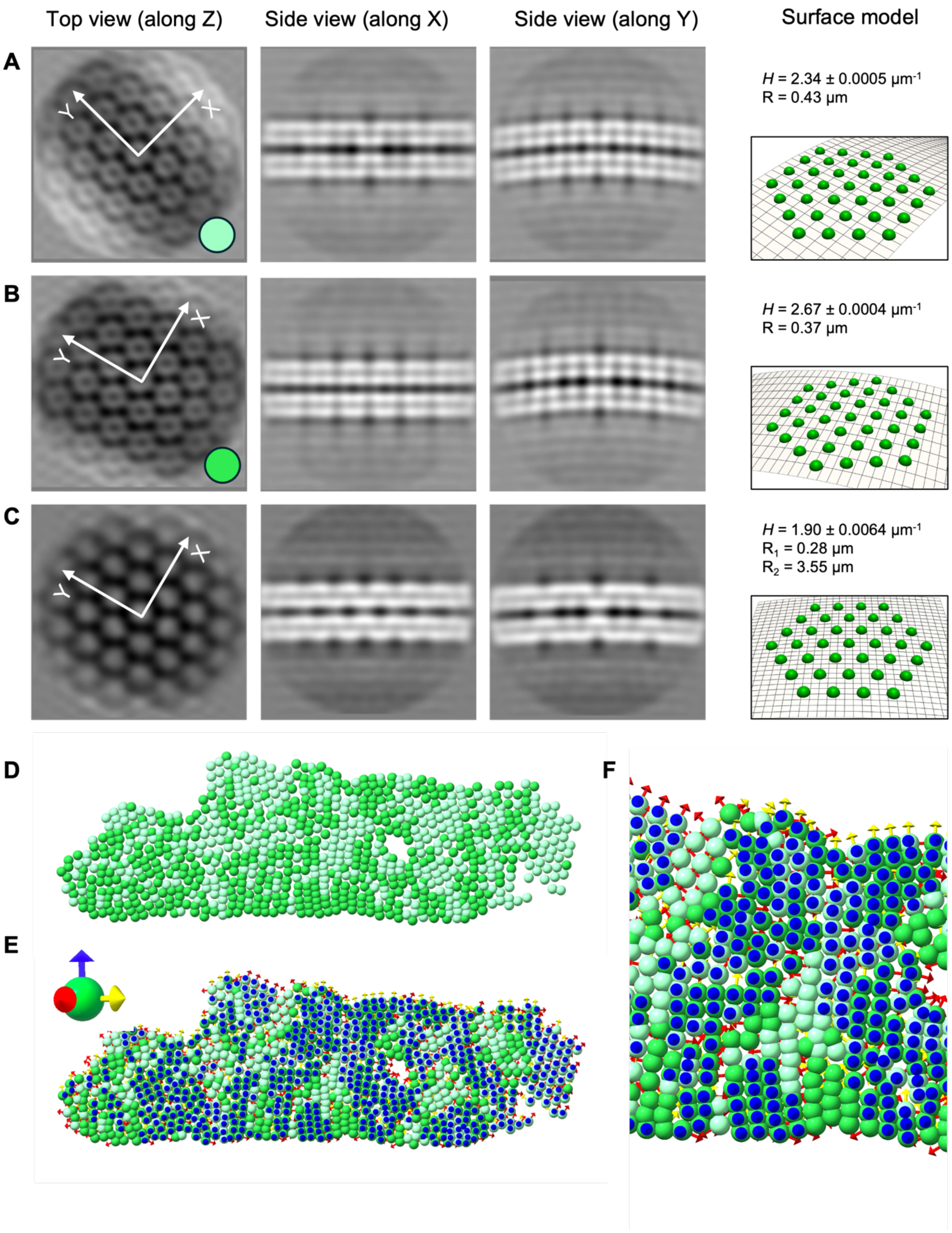

Top view (along Z)
Side view (along X)
Side view (along Y)
Surface model
A
H = 2.34 ± 0.0005 μm^{-1}
R = 0.43 μm
B
H = 2.67 ± 0.0004 μm^{-1}
R = 0.37 μm
C
H = 1.90 ± 0.0064 μm^{-1}
R_1 = 0.28 μm
R_2 = 3.55 μm
D
E
F

SFigure 5. Curvature in the connexin-43 lattice. (**A–C**) Cross-sections for three different class averages of gap junction channel (GJC) lattices are shown along the axes indicated. For each, a geometrical model is shown with a surface through the centre of the lattice with the positions of the GJCs (green). The first two lattices were fit to a cylinder surface (Gaussian curvature, K = 0) while the third was fitted with a quadratic surface (K = 9.86 nm^{-2}). Mean curvature at the centroid (*H*) and the corresponding characteristic radii (R, R_1, R_2) are reported. (**D**) The positions of the two classes (light green and green) plotted back on one tomogram. (**E**) The same view as in *D*, showing the orientation of each particle. The particles where the blue marker is away from the viewer have a different sign of curvature compared to those where the blue marker is toward the viewer. (**F**) A close-up of *E*.

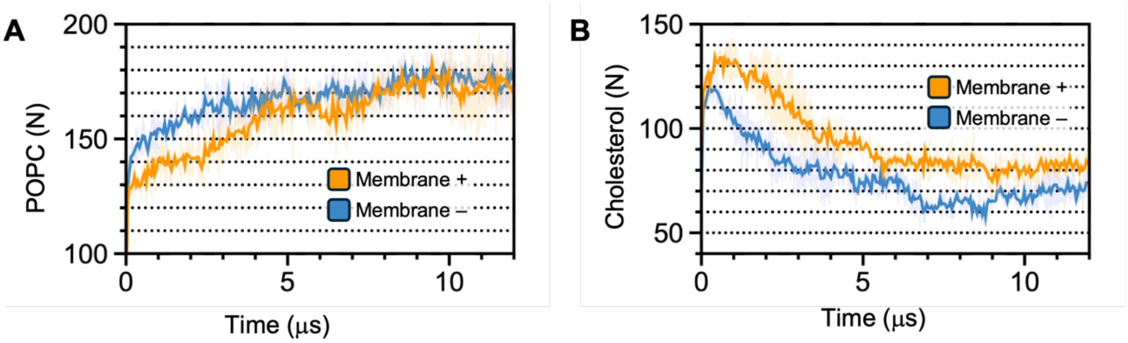

SFigure 6. Coarse-grained molecular dynamics of complex membranes around gap junction channels. (**A–B**) Simulation results for membranes containing POPC, CHOL, POPE, and sphingomyelin in a 30:30:20:20 ratio. The number of POPC lipids (*A*) and cholesterol molecules (*B*) around the centremost gap junction channel averaged from three independent simulations (N=3) is plotted as a function of the simulation time for both of the membranes (+ and – sides). The shaded area indicates the standard deviation.

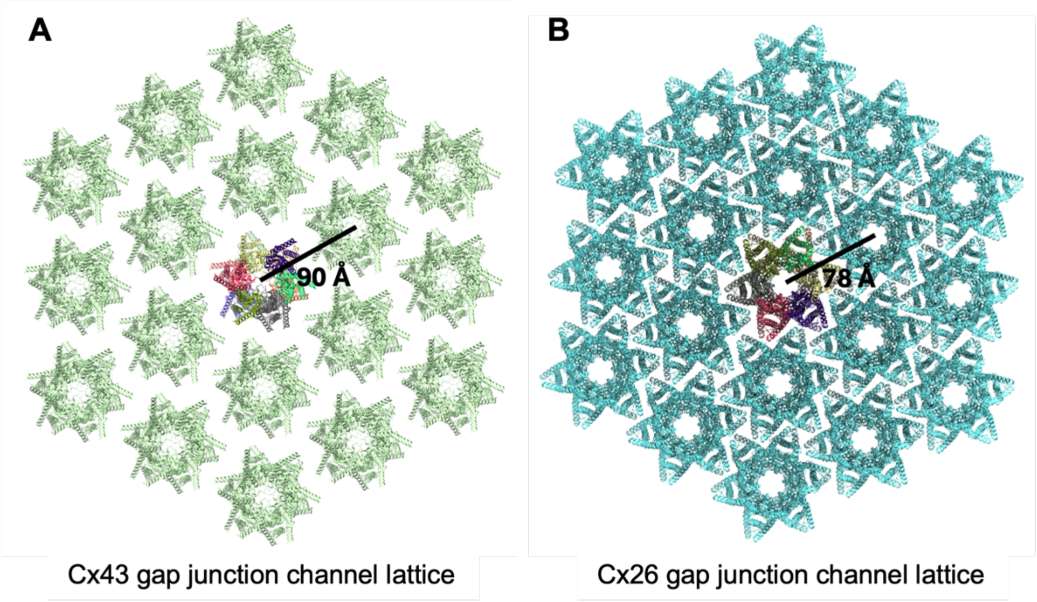

SFigure 7. Comparison of connexin-43 and connexin-26 lattices. (**A**) Connexin-43 (Cx43) gap junction channel (GJC) lattice, created by fitting a cryo-EM structure (PDB:7Z22) to the sub-tomogram reconstruction of the Cx43 GJC lattice patch. (**B**) A hypothetical model of connexin-26 (Cx26) GJC lattice, created by superposing a predicted structure of the full-length Cx26 GJC on the Cx43 GJC lattice and then adjusting the channel–channel distance to the known range (77±5 Å), avoiding clashes.

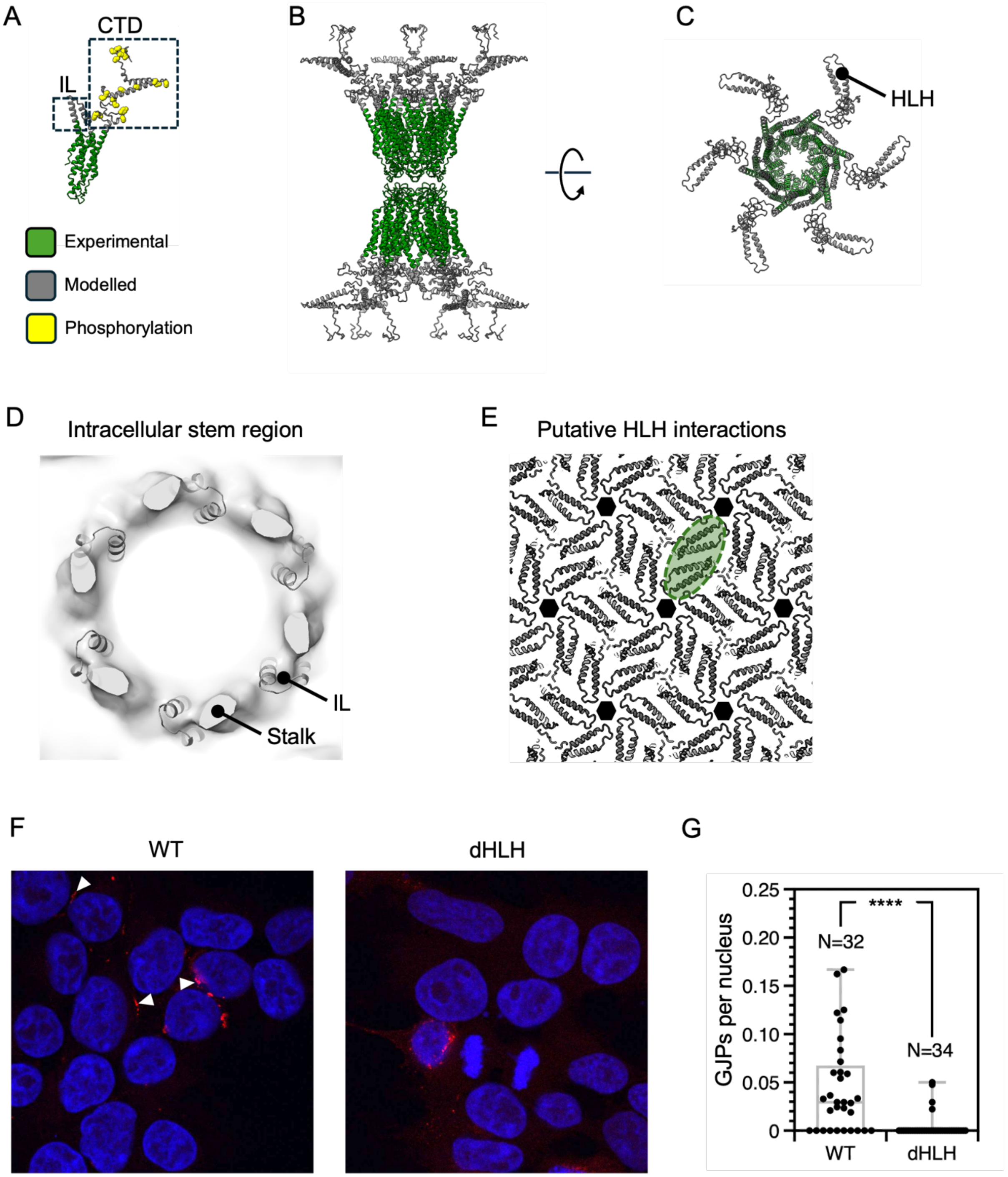

SFigure 8. Predicted structure of full length connexin-43 and the role of the putative helix-loop-helix motif. (**A**) The predicted model of the full length human connexin-43 (Cx43) is shown. The regions resolved in a cryo-EM structure (PDB:7Z22) are in green. The intracellular loop (IL)

and the C-terminal domain (CTD) regions present solely in the predicted structure are in grey. The phosphorylation sites are in yellow. (**B–C**) The predicted structure of the full-length human Cx43 gap junction channel is shown from the side (*B*) and from the top (*C*). The colouring is as in *a*. One helix-loop-helix (HLH) motif is indicated. The phosphorylation sites have been omitted for visual clarity. (**D**) An isosurface rendering of the intracellular stem region is shown with a transparent surface. One intracellular loop (IL) and one stalk region are indicated. (**E**) HLH motifs suggested to create the channel–channel contacts in the lateral contacts layer are shown. The local six-fold axes of symmetry are labelled with hexagons. One putative dimer of two HLHs is circled. (**F**) Representative fluorescence microscopy images of stable HEK293 cells expressing Cx43-SpyTag (WT) or the same construct lacking the putative helix-loop-helix motif (dHLH). Nuclei are stained with DAPI (blue), and Cx43 molecules are stained in red using SpyCatcher-AlexaFluor647. Gap junction plaques are labelled with white arrowheads. (**G**) Each dot represents GJPs normalised to the nucleus count from one randomly acquired image (WT, $n = 32$; dHLH, $n = 34$). Boxes indicate the median and interquartile range, with whiskers showing the full data range. Statistical significance was assessed using a two-tailed Mann–Whitney U test; ****, $p < 0.00001$.
